# Genomic organisation of the seven ParaHox genes of coelacanths

**DOI:** 10.1002/jez.b.22513

**Published:** 2013-06-17

**Authors:** John F Mulley, Peter WH Holland

**Affiliations:** 1 School of Biological Sciences, Bangor UniversityBangor, Gwynedd, United Kingdom; 2 Department of Zoology, University of OxfordOxford, United Kingdom

## Abstract

Human and mouse genomes contain six ParaHox genes implicated in gut and neural patterning. In coelacanths and cartilaginous fish, an additional ParaHox gene exists—*Pdx2*—that dates back to the genome duplications in early vertebrate evolution. Here we examine the genomic arrangement and flanking genes of all ParaHox genes in coelacanths, to determine the full complement of these genes. We find that coelacanths have seven ParaHox genes in total, in four chromosomal locations, revealing that five gene losses occurred soon after vertebrate genome duplication. Comparison of intergenic sequences reveals that some *Pdx1* regulatory regions associated with development of pancreatic islets are older than tetrapods, that *Pdx1* and *Pdx2* share few if any conserved non-coding elements, and that there is very high sequence conservation between coelacanth species.

The ParaHox gene cluster, first described in the cephalochordate amphioxus (Brooke et al., 1998), contains three tandemly arrayed homeobox genes (Gsx, Xlox, and Cdx) and dates to the base of the Bilateria (Ferrier and Minguillón, 2003) or earlier (Hui et al., 2008; Mendivil Ramos et al., 2012). The gene cluster has undergone gene losses, rearrangements, and duplications in different animal lineages. The ParaHox genes of bilaterian animals are involved in several developmental processes, notably in gut and neural patterning. Thus, endodermal midgut expression has been described for Xlox genes in vertebrates, amphioxus, echinoderm, annelids, and molluscs, with functional importance in gut formation demonstrated for sea urchin (Arnone et al., 2006) and for the well-studied vertebrate Xlox gene *Pdx1* (Jonsson et al., 1994; Offield et al., 1996; Brooke et al., 1998). The Cdx (*caudal-type homeobox*) genes are expressed posteriorly in many animals, and are important for development of the posterior gut and anus, as well as the posterior neural tube in insects and vertebrates (Moreno and Morata, 1999; Reece-Hoyes et al., 2002). Gsx (*genomic screen homeobox*, also known as *Gsh* or *ind*) genes are primarily involved in brain rather than gut patterning in mammals (Hsieh-Li et al., 1995; Valerius et al., 1995), although expression has been reported in the endocrine pancreas (Rosanas-Urgell et al., 2005). In at least some molluscs and annelids, the Gsx gene is expressed in the mouth region in addition to some nerve cells (Kulakova et al., 2008; Hui et al., 2009; Samadi and Steiner, 2010), consistent with the hypothesis that bilaterian ParaHox genes may have ancestrally patterned mouth, midgut, and anus (Holland, 2001).

Genome duplication events early in vertebrate evolution resulted in an increase in the number of ParaHox genes, such that the human genome has six ParaHox genes at four chromosomal locations. A cluster of three genes (*GSX1*, *PDX1*/*IPF1*, *CDX2*) is located at 13q12.1, but there are no other linked pairs or clusters of ParaHox genes in humans. Instead, three individual genes (*GSX2*, *CDX1*, *CDX4*) are located at three different genomic locations; analysis of flanking genes shows that these are remnants from three degenerate ParaHox gene clusters (Fig. 1). Humans have, therefore, lost six putative ParaHox genes in evolution, but the timing of this loss has been unclear.

**Figure 1:**
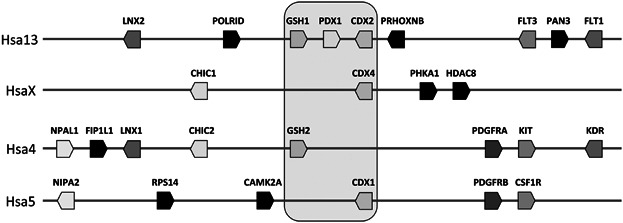
Organisation of ParaHox gene-containing regions in the human genome. An intact cluster of Gsx, Pdx, and Cdx genes in located on chromosome 13 (cluster A), with other ParaHox genes on the X chromosome (cluster B), chromosome 4 (cluster C), and chromosome 5 (cluster D).

A similar complement of ParaHox genes has been found in other jawed vertebrates examined, with three exceptions identified to date. First, zebrafish and pufferfish have lost *cdx2*, but have duplicates of *cdx1* (Mulley et al., 2006). The two *cdx1* genes in teleost fish are a remnant of the additional genome duplication that occurred in teleost fish evolution, though intriguingly this implies that duplicates of (at least) *gsx1*, *gsx2*, *pdx1*, and *cdx4* were lost, alongside two *cdx2* copies. Second, an ancient *Pdx2* gene was recently found in chondrichthyan fish (dogfish and skate), genomically linked to *Gsx2*; this implies that *Pdx2* was present in the common ancestor of extant jawed vertebrates (Mulley and Holland, 2010). Third, and most surprisingly, a *Pdx2* gene was also discovered in the coelacanths, lobe-finned fish that lie within the sarcopterygian clade of vertebrates, close to tetrapods (Mulley and Holland, 2010). The implication is that *Pdx2* existed for much of vertebrate evolution, at least up to the radiation of the sarcopterygians, and was lost independently in ray-finned fish and tetrapods.

The identification of this additional ParaHox gene, *Pdx2*, in coelacanths raised the question of whether other ParaHox genes might have survived for a significant length of time in vertebrate evolution, to be lost later in tetrapods. Coelacanths are sometimes referred to as “living fossils,” meaning that they have retained a relatively unchanged body plan for hundreds of millions of years, and it would be intriguing if their genome also retained ancient genes (such as *Pdx2*) lost in other lineages (Noonan et al., 2004; Powers and Amemiya, 2004; Amemiya et al., 2010). To investigate this, we sequenced and assembled the genomic regions flanking all ParaHox genes in a coelacanth, and their bordering non-homeobox genes, to define the limits of each ParaHox gene cluster or gene cluster remnant. We find a surprising level of sequence conservation between the extant coelacanth species and demonstrate the absence of any additional ParaHox genes beyond the known seven. We also show that known regulatory regions associated with the *Pdx1* gene are older than previously thought and that the paralogous *Pdx1* and *Pdx2* genes of coelacanths do not appear to share regulatory architecture.

## RESULTS

**Figure 2:**
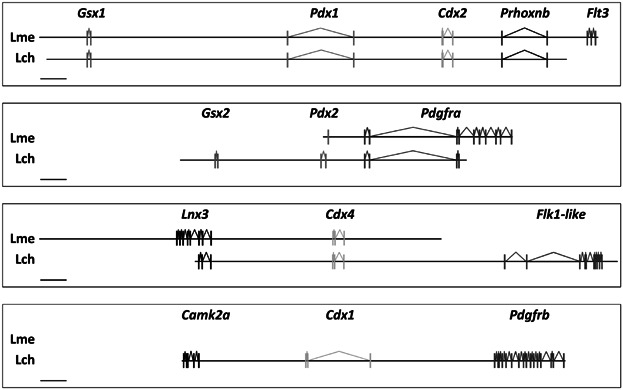
Organisation of ParaHox genes in the two extant species of Coelacanth (Lme—Indoenesian coelacanth *Latimeria menadoensis*, Lch—African coelacanth *Latimeria chalumnae*). Lme data is based on sequenced BAC clones and Lch data is derived from genome sequence information. The complement of ParaHox genes in both species is two Gsx (*Gsx1*, *Gsx2*), two Xlox (*Pdx1*, *Pdx2*), and three Cdx (*Cdx1*, *Cdx2*, *Cdx4*). Scale bar is 10 kb.

**Figure 3:**
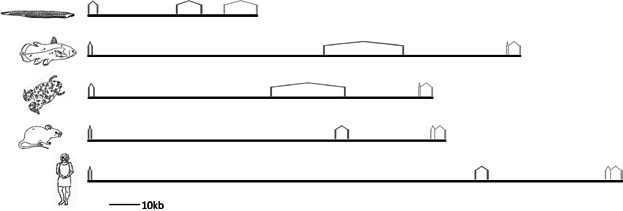
Genomic organisation of the ParaHox A cluster in vertebrates. Vertebrate ParaHox A clusters are much larger than the amphioxus cluster, although the relative spacing of genes is conserved. From top; amphioxus (*Branchiostoma floridae*), Indonesian coelacanth (*Latimeria menadoensis*), frog (*Xenopus tropicalis*), Mouse (*Mus musculus*), and human (*Homo sapiens*).

We previously reported that coelacanths have retained an additional ParaHox gene (*Pdx2*) that has been independently lost in other bony vertebrate lineages (Mulley and Holland, 2010) and set out to discover whether they may also have retained additional members of the Gsx, Xlox and Cdx gene families. Sequencing and assembly of two overlapping BAC clones from Indonesian coelacanth yielded a 217,497 bp contiguous sequence containing the full coding sequences of the ParaHox genes *Gsx1*, *Pdx1*, and *Cdx2*, plus complete or partial sequences of the flanking *Prhoxnb* (*ParaHox cluster neighbour*) and *Flt3* (*FMS-like tyrosine kinase 3*) genes (Fig. 2). A 154,768 bp *Pdx2*-containing Indonesian coelacanth BAC clone has been described previously (Mulley and Holland, 2010) and also contains the *Pdgfrα* (*Alpha-type platelet-derived growth factor receptor*) gene. A fourth ParaHox-containing BAC clone of 185,347 bp assembled into six ordered pieces and contained the *Cdx4* and *Lnx3* (*Ligand of numb protein X 3*) genes. It has been suggested that the ratio of the relative spacing of genes (Gsx to Xlox, and Xlox to Cdx) is conserved in chordate ParaHox clusters (Ferrier et al., 2005); we find that this pattern is broadly conserved in coelacanths (Fig. 3) (see Table S1, Supplementary information).

Evidence for ParaHox genes and their genomic neighbours from these sequenced BAC clones, as well as extensive degenerate PCR experiments (using a variety of primer combinations and reaction conditions), plus analysis of preliminary whole genome sequence information available for the African coelacanth *Latimeria chalumnae*, demonstrates that the complement of ParaHox genes in coelacanths is: two members of the Gsx gene family (*Gsx1*, *Gsx2*); two members of the Xlox gene family (*Pdx1* and *Pdx2*) and three members of the Cdx gene family (*Cdx1*, *Cdx2*, *Cdx4*) (Fig. 2). We found no evidence for additional ParaHox genes in coelacanths and PCR-based analyses have so far failed to identify any others in other vertebrate groups, including cartilaginous fish (Mulley and Holland, 2010) and lungfish (*Neoceratodus forsteri*, data not shown). We suggest that the situation in coelacanths is representative of the basal jawed vertebrate condition.

The availability of sequence information for the canonical ParaHox A cluster (*Gsx1*, *Pdx1*, *Cdx2*) and the *Gsx2*–*Pdx2* cluster (cluster C) enables comparisons of the conserved non-coding (putative regulatory) sequences associated with the paralogous Pdx genes—something that is desirable given the important role played by the *Pdx1* gene in pancreas development (Jonsson et al., 1994; Offield et al., 1996; Stoffers et al., 1997; Schwitzgebel et al., 2003) and in glucose-responsive regulation of insulin gene expression (Ohlsson et al., 1993), and its relevance to diabetes (Cockburn et al., 2004; Hani et al., 1999; Macfarlane et al., 1999). These comparisons were not previously possible with the fragmentary data from earlier studies and shed light on the evolutionary history of pancreas development and insulin regulation.

**Figure 4:**
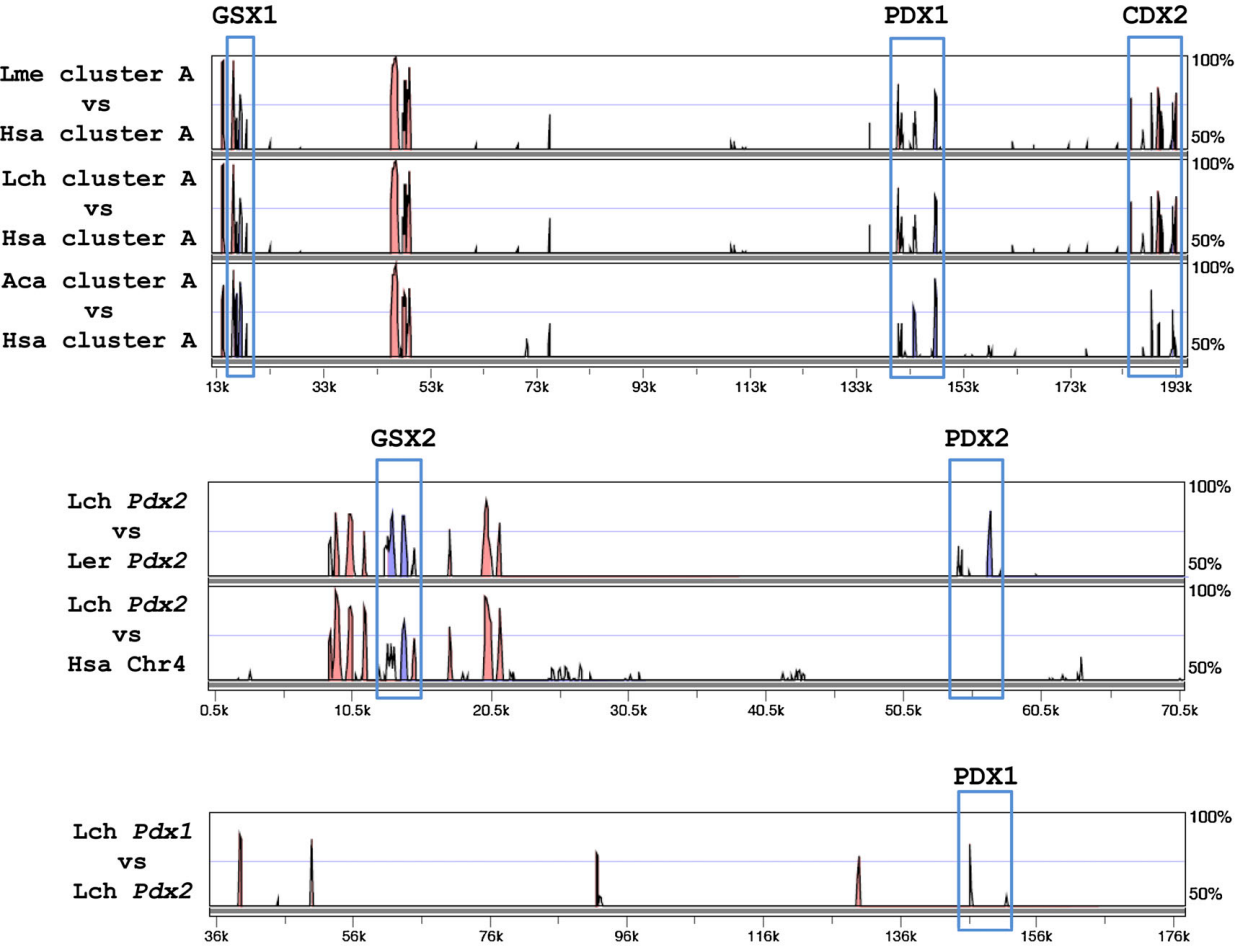
mVISTA alignments of vertebrate ParaHox regions. Top: Alignment of Indonesian coelacanth (Lme), African coelacanth (Lch), and Bowfin (Aca) A clusters against the human A cluster; middle—Alignment of Little skate (Ler) and human (Hsa) C clusters against the African coelacanth C cluster; bottom—Alignment of clusters A and C in African coelacanth (Lch). The conserved non-coding sequences in the bottom pane share high sequence similarity with non-coding sequences in other Coelacanth BAC clones and therefore likely represent a Coelacanth-specific repetitive element.

We first used mVISTA (Frazer et al., 2004) to compare the human, Bowfin (*Amia calva*—a basal ray-finned fish with an intact cluster (Mulley et al., 2006)) and African and Indonesian coelacanth ParaHox A clusters to identify conserved non-coding regions of DNA (Fig. 4). This revealed a pattern of conservation within and around the canonical ParaHox cluster similar to that in other vertebrates (Mulley et al., 2006). In contrast, no conserved non-coding elements were identified between the Coelacanth *Pdx1* and *Pdx2* regions, apart from a short region with no similarity to known *Pdx1* enhancer sequences and with high sequence conservation to other coelacanth BAC clones in Genbank, suggesting a repetitive element in the Coelacanth genome. We find no conserved non-coding elements between the *Pdx2*-containing regions of *L. chalumnae* and Little Skate (*Leucoraja erinacea*); the most highly conserved elements are close to *Gsx2* and also conserved in the human genome (which lacks *Pdx2*), suggesting they regulate *Gsx2*. To assess if the evolutionary rates of *Pdx1* and *Pdx2* were unequal, we conducted relative rate tests. No significant differences were detected (*L. chalumnae P* = 0.76808; *Scyliorhinus canicula P* = 0.54649).

The human and rodent *Pdx1* genes have been intensively studied and a number of regions important for transcriptional regulation have been identified, some of which are conserved in other tetrapods (Gerrish et al., 2000, 2004; Boyer et al., 2006; Fujitani et al., 2006; Miyatsuka et al., 2007; Wiebe et al., 2007). We analyzed DNA sequence 5′ of the coelacanth *Pdx1* coding regions, searching for evidence of Areas I–III (involved in differentiation and maintenance of pancreatic islets (Fujitani et al., 2006)), Area IV (involved in driving gene expression specifically in β-cells (Gerrish et al., 2004)) and the E-box (involved in β-cell-specific expression of *Pdx1* (Melloul et al., 2002)). We find sequence 5′ to coelacanth *Pdx1* gene corresponding to Area I (−2,761 to −2,457 in human), Area II (−2,153 to −1,923 in human), and Area III (−1,879 to −1,600 in human) (see Figs. S1–S3 for alignments). We found no evidence for Area IV −8,656 to −8,155 (in human) or the E-box (−104 in human) in the proximal promoter region.

The extent of sequence conservation between the two extant coelacanth species was also examined. Analyses of ParaHox genes for which complete gene sequence was available from both species (*Gsx1*, *Pdx1*, *Cdx2*, *Cdx4*) revealed that coding sequences were 100% conserved, whilst introns were typically >99% identical (Gsx1 intron, 99.8; Pdx1 intron, 99.6; Cdx2 introns, 99.1; Cdx4 introns, 99.8). Furthermore, a larger comparison across the entire ParaHox A cluster (from the start codon of *Gsx1* to the start of *Cdx2*, comprising over 120 kb; discounting regions with Ns in *L. chalumnae*) revealed >99% similarity. These findings are in line with those reported elsewhere and show that the ParaHox cluster is conserved at a similar level to the Hox clusters of the two coelacanth species, and slightly more highly conserved than some other genomic regions (see Amemiya et al., 2013; Table S11). This is an extremely high level of sequence similarity between the two species, considering the suggestion that they diverged 24–44 million years ago (Inoue et al., 2005). By comparison, New World monkeys (Platyrrhini) and Old World monkeys and apes (Catarrhini) diverged ∼35 million years ago (Schrago and Russo, 2003) and our analysis of sequence conservation between human and Common Marmoset (*Callithrix jacchus*) ParaHox A clusters (>150 kb) showed 66% sequence similarity.

## DISCUSSION

The presence of seven ParaHox genes in coelacanths lends support to a model for ParaHox gene evolution involving whole genome duplications (WGDs) early in vertebrate ancestry followed by gene losses. It suggests that the seven ParaHox gene state found previously in cartilaginous fish (Mulley and Holland, 2010) and now also demonstrated in coelacanths was established rapidly following WGD. Analyses of the genes flanking the coelacanth ParaHox clusters (see Figs. 1 and 2) identifies several other gene families that were duplicated via WGD (*Platelet-derived growth factor receptor*, *Ligand of Numb Protein X*, *FMS-like tyrosine kinase*) and show conserved synteny to human and other vertebrate ParaHox clusters. The apparent slow rate of molecular evolution and genomic rearrangement in cartilaginous fish and coelacanths (Mulley and Holland, 2010; Wang et al., 2009) suggests that both may be excellent models for the study of events post-WGD and later genomic re-organization in the actinopterygian and sarcopterygian lineages.

Comparison of coelacanth ParaHox cluster sequences to known *Pdx1* regulatory regions from mammals (Melloul et al., 2002; Gerrish et al., 2004; Fujitani et al., 2006) reveals that several regulatory elements are more ancient than previously thought (see alignments in Supplementary information). Our results also suggest that the loss of Area II in birds is potentially unique to that lineage (or possibly unique to reptiles, as we have also not been able to find any similarity to this region in the sequence upstream of the Green Anole (*Anolis carolinensis*) *Pdx1* gene in the AnoCar2.0 genome assembly). In Areas I–III, the highest level of sequence conservation was seen in only part of the region previously defined (Gerrish et al., 2000) and it is possible that these areas represent core enhancer regions that have been elaborated on later in tetrapod or mammalian evolution, as has been reported for lamprey conserved non-coding sequences (McEwen et al., 2009). Our findings from the coelacanths may be particularly useful for future studies of these sites, as many of the regions that are 100% conserved are around 8–10 bp in length, similar to the size of most transcription factor binding sites. Surprisingly, we were unable to find any conserved non-coding sequences between the *Pdx1* and *Pdx2* gene-containing regions of coelacanths suggesting that these genes do not share regulatory architecture. We were also unable to identify any conserved non-coding sequences between the *Pdx2*-containing regions of coelacanth and Little skate, suggesting there may have been differential loss of regulatory elements following WGD. Given the difficulty in accessing tissue samples from the CITES-listed coelacanths, it is unlikely that we will be able to discover the function of the *Pdx2* gene in this lineage. Model cartilaginous fish such as the Little skate (*L. erinacea*) or Lesser Spotted Catshark (*S. canicula*), both of which have ongoing transcriptome and whole genome sequencing projects, offer the best chance to determine the role of the *Pdx2* gene in embryos and adults.

## MATERIALS AND METHODS

### Isolation of Indonesian Coelacanth (*Latimeria menadoensis*) ParaHox Genes by Degenerate PCR

Homeobox-containing fragments of members of the Gsx, Xlox, and Cdx gene families were isolated from genomic DNA using the following degenerate PCR primers: for Gsx JMGsx1a: 5′-ATG YCG MGV TCY TTY YWB GT-3′ (forward); JMGsx1b: 5′-GTN GAY TCN YTV ATN WTN ARG GA-3′ (nested forward); Gsx3: 5′-TTG CCY TCY TTY TTG TGC TT-3′ (reverse); GsxSO2: 5′-CAN CKD CGR TTY TGR AAC CA-3′ (nested reverse); for Cdx JmCdx: 5′-GGN AAR CAN MGR ACV AAR GA-3′ (forward); CdxSO1: 5′-CTRGARCTGGARAARGARTT-3′ (nested forward); CdxSO2: 5′-NVK NVK RTT YTG RAA CCA-3′ (reverse) and for Pdx JMXloxIc: 5′-GAC GAC AAC AAG MGN CAN AGR AC-3′ (forward); Xlox2: 5′-CAG CTG CTV GAG CTV GAG AA-3′ (nested forward); Xlox3: 5′-YTC CTC YTT YTT CCA CTT CAT-3′ (reverse); XSO2: 5′-GCG NCG RTT YTG GAA CCA GAT-3′ (nested reverse). The resulting homeobox fragments (representing two Gsx genes, two Pdx genes, and three Cdx genes) were digoxigenin (DIG)-labeled and used to screen a high coverage BAC library for Indonesian coelacanth (from the Genome Resource Centre, Benaroya Research Institute, Seattle, WA, USA (Danke et al., 2004)). Positive clones were verified by PCR prior to sequencing and were found to contain *Pdx1*-like and *Cdx2*-like gene fragments (Clones 95G3 and 161J8), a *Pdx2*-like fragment (Clone 188I4) and a *Cdx4*-like fragment (Clone 52G17). No *Cdx1*-positive clones were found during this library screening, nor were they identified using a variety of DIG-labelled homeobox probes. As previously reported (Mulley and Holland, 2010), clone 188I4 containing *Pdx2* (accession HM134895) was sequenced to 9.7× coverage using Sanger sequencing (performed at the Washington University Genome Centre, St. Louis, MI) and clones 52G17 (*Cdx4*) and 95G3 (*Pdx1*) were sequenced in the same way to 10.1× coverage and 6.0× coverage respectively. Clone 161J8 was sequenced to ∼40× coverage using Roche 454 GS FLX Titanium technology (performed at the Centre for Genomic Research, University of Liverpool, UK). Genes were predicted using BLAST and GenScan and by alignment to known orthologous genes from other vertebrates. BAC clone sequences are deposited in GenBank under accession numbers KC914566–KC914567.

### Phylogenetic Analysis

Orthology of genes encoded by the sequenced BAC clones was confirmed using phylogenetic analysis. Amino acid sequences were aligned using ClustalX (Larkin et al., 2007) and edited by eye to maximize contiguity of alignable sequence; maximum likelihood phylogenetic trees were constructed with PhyML (Guindon and Gascuel, 2003) using the JTT matrix and 1,000 bootstrap replicates. All nodes had >50% support and trees were rooted with amphioxus (*Branchiostoma floridae*) or fly (*Drosophila melanogaster*) sequences. The resulting trees are provided as Supplementary information. Tajima's Relative Rate Test (Tajima, 1993) was conducted on amino acid sequences in *MEGA* version 5 (Tamura et al., 2011) using amphioxus Xlox as an outgroup. All positions containing gaps were eliminated.

### Coelacanth Genome Survey and Comparative Genomics

Genomic data for the African coelacanth (*L. chalumnae*) became available in late 2011 and was used to fill gaps in our *L. menadoensis* BAC clone data. BLAST surveys using *L. menadoensis* genes were carried out against the LatCha1 September 2011 v64 assembly at PreEnsembl (http://pre.ensembl.org) and comparisons for sequence conservation were carried out using mVISTA (Frazer et al., 2004) and the AVID alignment program (Bray et al., 2003) using a 100 bp window 70% conservation level. Additional analyses of conserved non-coding sequences and pairwise alignments of *L. menadoensis* and *L. chalumnae* sequences were carried out using ClustalW (Larkin et al., 2007) implemented in BioEdit (Hall, 1999). Common Marmoset (*C. jacchus*) genome sequence analyses were based on CalJac3.2.1 (Release 58, May 2010) data from Ensembl—www.ensembl.org/Callithrix_jacchus).

## Supporting Information

**Figure S1.** Alignment of Area I of the *Pdx1* promoter, based on sequence at −2,761 to −2,457 upstream of the human *PDX1* gene. Hsa—human; Mmu—mouse; Gga—chicken; Xtr—frog; Lme—Indonesian Coelacanth; Lch—African Coelacanth. **Figure S2.** Alignment of Area II of the *Pdx1* promoter, based on sequence at −2,153 to −1,923 upstream of the human *PDX1* gene. Hsa—human; Mmu—mouse; Xtr—frog; Lme—Indonesian Coelacanth; Lch—African Coelacanth. **Figure S3.** Alignment of Area III of the *Pdx1* promoter, based on sequence at −1,879 to −1,600 upstream of the human *PDX1* gene. Hsa—human; Mmu—mouse; Gga—chicken; Lme—Indonesian Coelacanth; Lch—African Coelacanth. **Figure S4.** Maximum likelihood tree of vertebrate Gsx genes. Hsa, *Homo sapiens*; Mmu, *Mus musculus*; Xtr, *Xenopus tropicalis*; Lch, *Latimeria chalumnae*; Lme, *Latimeria menaodensis*; Dre, *Danio rerio*; Tru, *Takifugu rubripes*; Tni, *Tetraodon nigroviridis*; Sca, *Scyliorhinus canicula*; Bfl, *Branchiostoma floridae*. **Figure S5.** Maximum likelihood tree of vertebrate Cdx genes. Hsa, *Homo sapiens*; Bta, *Bos taurus*; Mdo, *Monodelphis domestica*; Mmu, *Mus musculus*; Aca, *Anolis caroliniensis*; Xtr, *Xenopus tropicalis*; Lch, *Latimeria chalumnae*; Lme, *Latimeria menaodensis*; Dre, *Danio rerio*; Tru, *Takifugu rubripes*; Tni, *Tetraodon nigroviridis*; Sca, *Scyliorhinus canicula*; Bfl, *Branchiostoma floridae*. **Figure S6.** *Maximum likelihood tree of vertebrate Pdgfr genes*. Hsa, *Homo sapiens*; Mmu, *Mus musculus*; Gga, *Gallus gallus*; *Lch*, *Latimeria chalumnae*; Lme, *Latimeria menaodensis*; Dre, *Danio rerio*; Tru, *Takifugu rubripes*; Tni, *Tetraodon nigroviridis*; Gac, *Gasterosteus aculeatus*; Drosophila, *Drosophila melanogaster*. **Figure S7.** *Maximum likelihood tree of vertebrate Prhoxnb genes*. Hsa, *Homo sapiens*; Mmu, *Mus musculus*; Xtr, *Xenopus tropicalis*; Lch, *Latimeria chalumnae*; Lme, *Latimeria menaodensis*; Dre, *Danio rerio*; Tru, *Takifugu rubripes*; Bfl, *Branchiostoma floridae*. **Table S1.** Relative spacing of ParaHox genes in vertebrates (Bfl, *Branchiostoma floridae*; Hsa, *Homo sapiens*; Mmu, *Mus musculus*; Lme, *Latimeria menadoensis*; Xtr, *Xenopus tropicalis*; Aca, *Amia calva*).
