# Supplementary material for: Genomic organisation of the seven ParaHox genes of coelacanths
Source: J Exp Zool B Mol Dev Evol. 2013 Jun 17;322(6):352–8. doi: 10.1002/jez.b.22513 (PMC4471637; doi:10.1002/jez.b.22513)
Supplement: Supplementary file 1 — Figure S1. Alignment of Area I of the Pdx1 promoter, based on sequence at −2,761 to −2,457 upstream of the human PDX1 gene. Hsa—human; Mmu—mouse; Gga—chicken; Xtr—frog; Lme—Indonesian Coelacanth; Lch—African Coelacanth. Figure S2. Alignment of Area II of the Pdx1 promoter, based on sequence at −2,153 to −1,923 upstream of the human PDX1 gene. Hsa—human; Mmu—mouse; Xtr—frog; Lme—Indonesian Coelacanth; Lch—African Coelacanth. Figure S3. Alignment of Area III of the Pdx1 promoter, based on sequence at −1,879 to −1,600 upstream of the human PDX1 gene. Hsa—human; Mmu—mouse; Gga—chicken; Lme—Indonesian Coelacanth; Lch—African Coelacanth. Figure S4. Maximum likelihood tree of vertebrate Gsx genes. Hsa, Homo sapiens; Mmu, Mus musculus; Xtr, Xenopus tropicalis; Lch, Latimeria chalumnae; Lme, Latimeria menaodensis; Dre, Danio rerio; Tru, Takifugu rubripes; Tni, Tetraodon nigroviridis; Sca, Scyliorhinus canicula; Bfl, Branchiostoma floridae. Figure S5. Maximum likelihood tree of vertebrate Cdx genes. Hsa, Homo sapiens; Bta, Bos taurus; Mdo, Monodelphis domestica; Mmu, Mus musculus; Aca, Anolis caroliniensis; Xtr, Xenopus tropicalis; Lch, Latimeria chalumnae; Lme, Latimeria menaodensis; Dre, Danio rerio; Tru, Takifugu rubripes; Tni, Tetraodon nigroviridis; Sca, Scyliorhinus canicula; Bfl, Branchiostoma floridae. Figure S6. Maximum likelihood tree of vertebrate Pdgfr genes. Hsa, Homo sapiens; Mmu, Mus musculus; Gga, Gallus gallus; Lch, Latimeria chalumnae; Lme, Latimeria menaodensis; Dre, Danio rerio; Tru, Takifugu rubripes; Tni, Tetraodon nigroviridis; Gac, Gasterosteus aculeatus; Drosophila, Drosophila melanogaster. Figure S7. Maximum likelihood tree of vertebrate Prhoxnb genes. Hsa, Homo sapiens; Mmu, Mus musculus; Xtr, Xenopus tropicalis; Lch, Latimeria chalumnae; Lme, Latimeria menaodensis; Dre, Danio rerio; Tru, Takifugu rubripes; Bfl, Branchiostoma floridae. Table S1. Relative spacing of ParaHox genes in vertebrates (Bfl, Branchiostoma floridae; Hsa, Homo sapiens; Mmu, Mu [file jez0322-0352-sd1.doc]

Supplementary Material

Supplemental Table S1. Relative spacing of ParaHox genes in vertebrates (Bfl, *Branchiostoma floridae*; Hsa, *Homo sapiens*; Mmu, *Mus musculus*; Lme, *Latimeria menadoensis*; Xtr, *Xenopus tropicalis*; Aca, *Amia calva*)

|  | Bfl | Hsa | Mmu | Lme | Xtr | Aca |
| --- | --- | --- | --- | --- | --- | --- |
| Intergenic distance (in bp)  [**Gsx**_(stop)_ - **Xlox**_(ATG start)_] | 25669 | 126190 | 80244 | 76045 | 58055 | 16076 |
| [**Xlox**_(stop)_ – **Cdx**_(stop)_] | 6947 | 38416 | 27017 | 33825 | 23123 | 7117 |
| Ratio (Gsx to Xlox : Xlox to Cdx) | 3.69:1 | 3.28:1 | 2.97:1 | 2.25:1 | 2.51:1 | 2.26:1 |


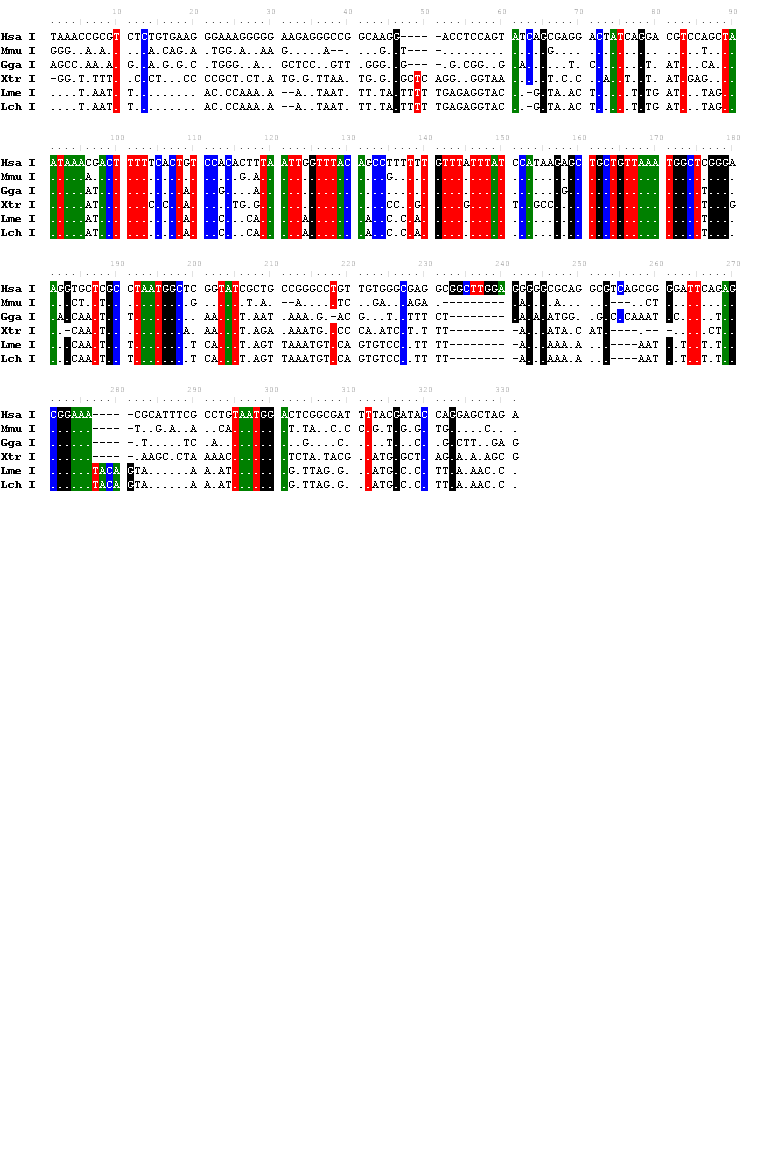


Supplemental figure S1 – Alignment of Area I of the *Pdx1* promoter, based on sequence at -2761 to -2457 upstream of the human *PDX1* gene. Hsa – human; Mmu – mouse; Gga – chicken; Xtr – frog; Lme – Indonesian Coelacanth; Lch – African Coelacanth.


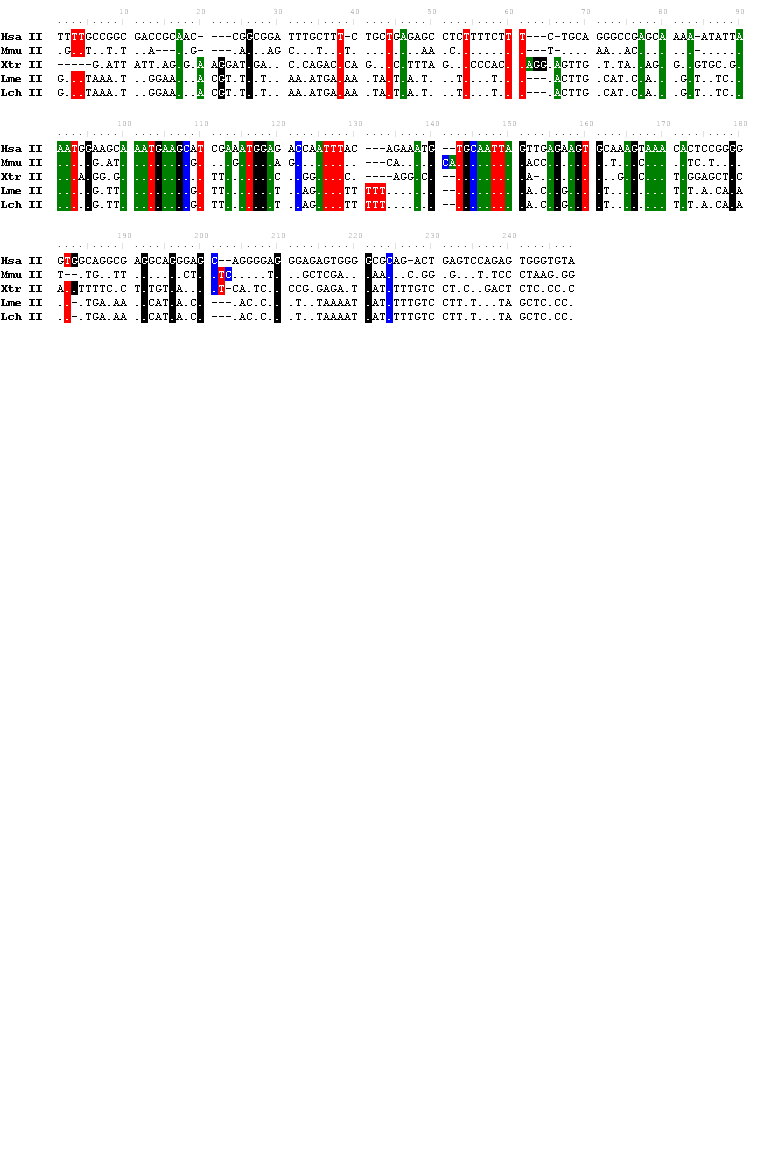


Supplemental figure S2 – Alignment of Area II of the *Pdx1* promoter, based on sequence at -2153 to -1923 upstream of the human *PDX1* gene. Hsa – human; Mmu – mouse; Xtr – frog; Lme – Indonesian Coelacanth; Lch – African Coelacanth.


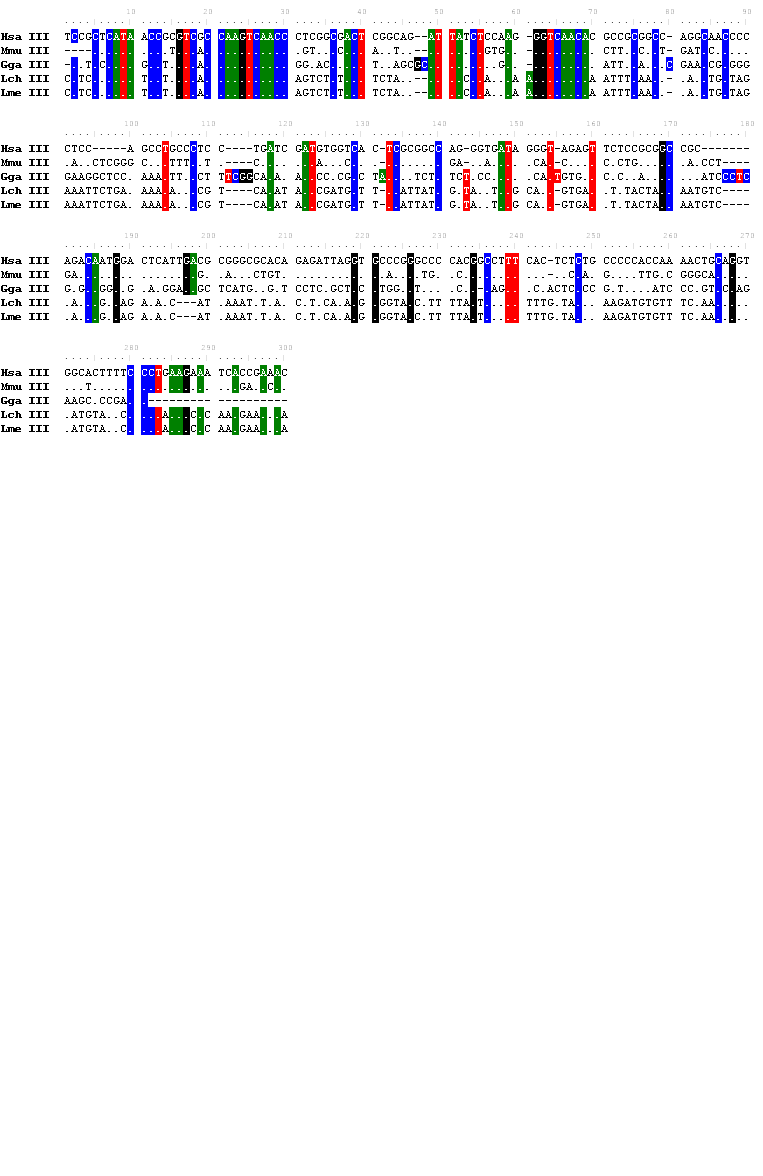


Supplemental figure S3 – Alignment of Area III of the *Pdx1* promoter, based on sequence at -1879 to -1600 upstream of the human *PDX1* gene. Hsa – human; Mmu – mouse; Gga – chicken; Lme – Indonesian Coelacanth; Lch – African Coelacanth.


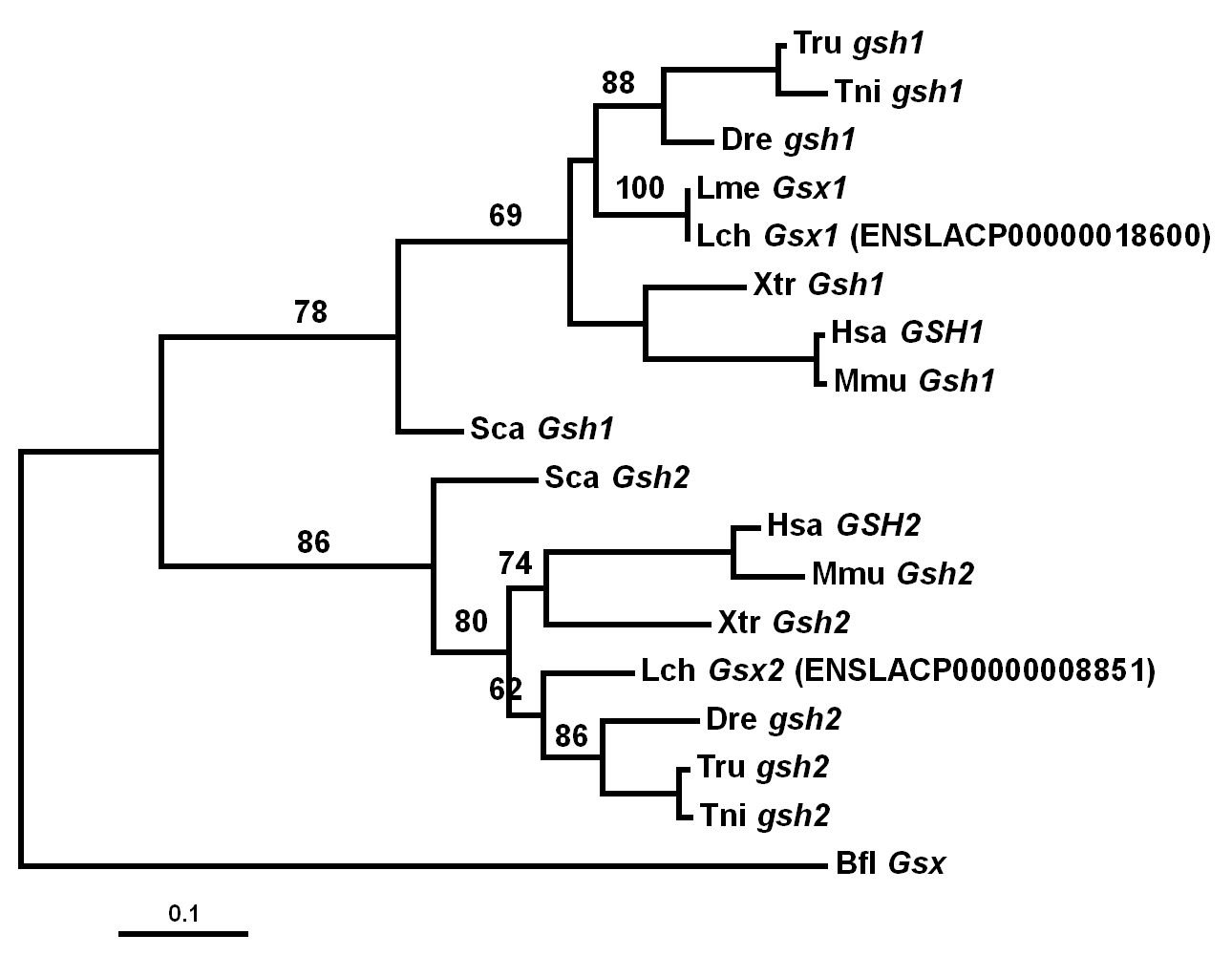


Supplemental figure S4. Maximum likelihood tree of vertebrate Gsx genes. Hsa, *Homo sapiens*; Mmu, *Mus* *musculus*; Xtr, *Xenopus tropicalis*; Lch, *Latimeria chalumnae*; Lme, *Latimeria menaodensis*; Dre, *Danio rerio*; Tru, *Takifugu rubripes*; Tni, *Tetraodon nigroviridis*; Sca, *Scyliorhinus canicula;* Bfl, *Branchiostoma floridae*


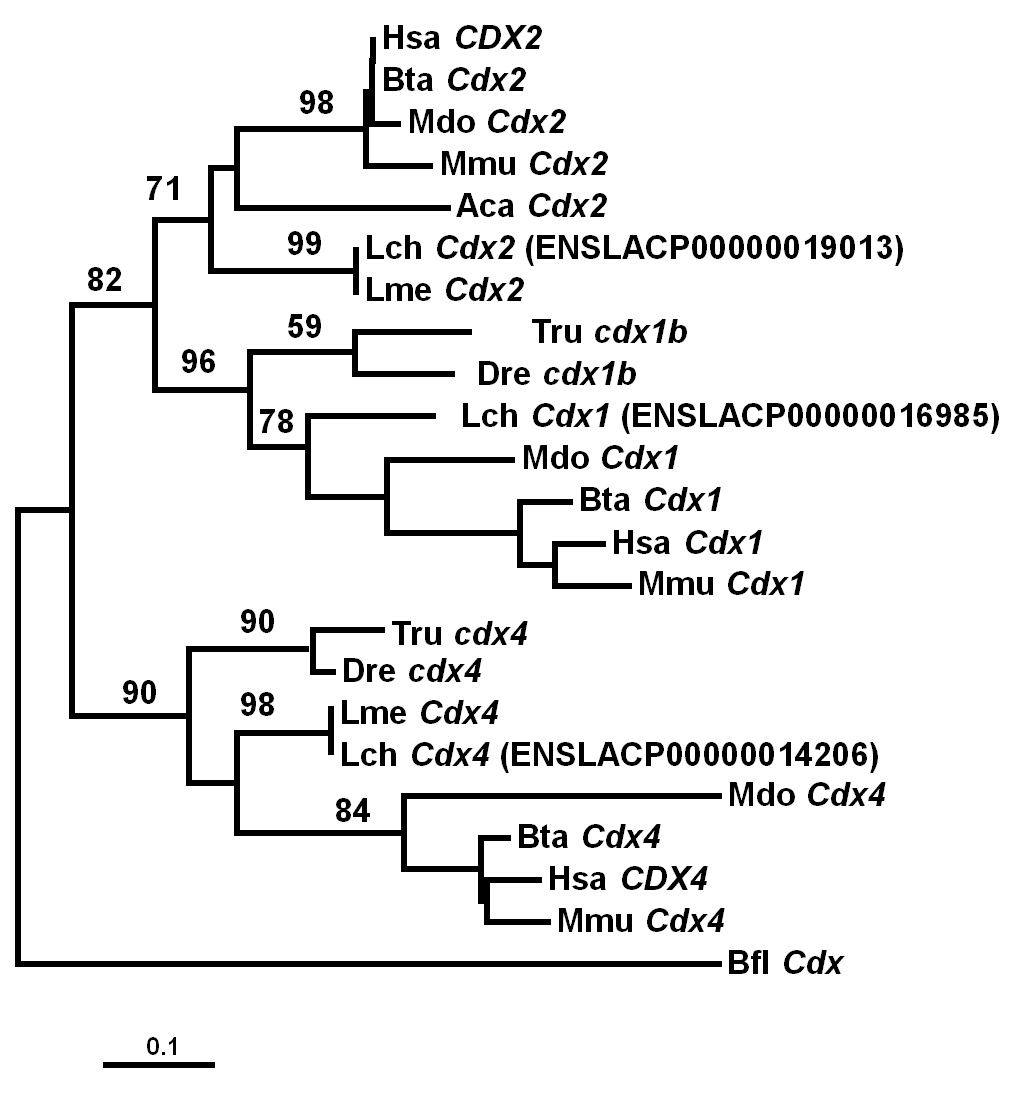


Supplemental figure S5. Maximum likelihood tree of vertebrate Cdx genes. Hsa, *Homo sapiens*; Bta, *Bos* *taurus*; Mdo, *Monodelphis domestica*; Mmu, *Mus* *musculus*; Aca, *Anolis caroliniensis*; Xtr, *Xenopus tropicalis*; Lch, *Latimeria chalumnae*; Lme, *Latimeria menaodensis*; Dre, *Danio rerio*; Tru, *Takifugu rubripes*; Tni, *Tetraodon nigroviridis*; Sca, *Scyliorhinus canicula;* Bfl*, Branchiostoma floridae*


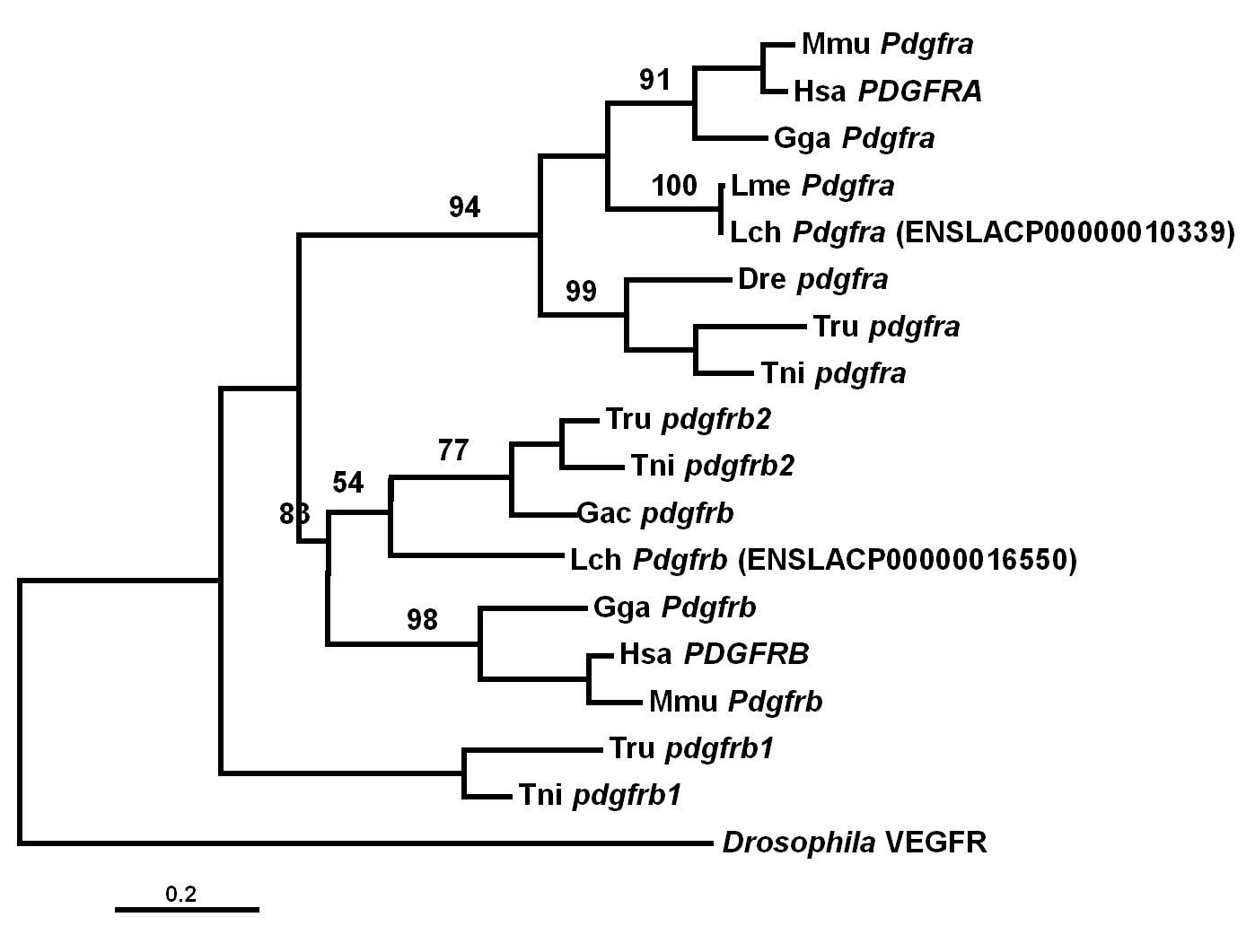


Supplemental figure S6. *Maximum likelihood tree of vertebrate Pdgfr genes.* Hsa*, Homo sapiens;* Mmu*, Mus musculus;* Gga*, Gallus gallus; Lch, Latimeria chalumnae;* Lme*, Latimeria menaodensis;* Dre*, Danio rerio;* Tru*, Takifugu rubripes;* Tni*, Tetraodon nigroviridis;*Gac, *Gasterosteus aculeatus*; Drosophila*, Drosophila melanogaster*


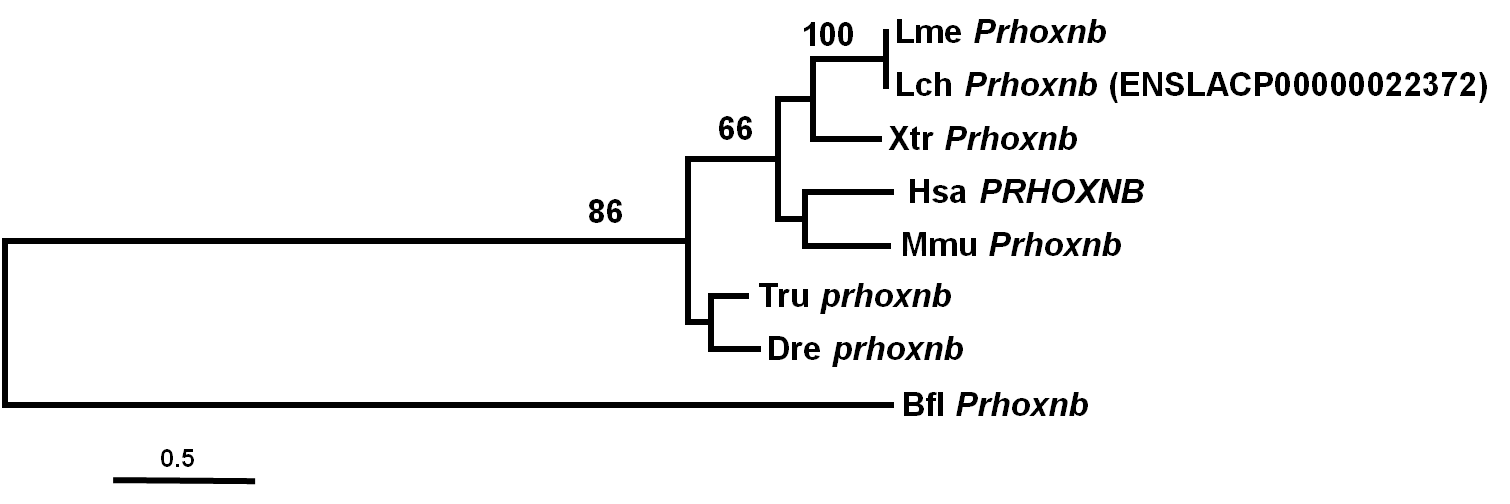


Supplemental figure S7. *Maximum likelihood tree of vertebrate Prhoxnb genes.* Hsa*, Homo sapiens;* Mmu*, Mus musculus;* Xtr*, Xenopus tropicalis;* Lch*, Latimeria chalumnae;* Lme*, Latimeria menaodensis;* Dre*, Danio rerio;* Tru*, Takifugu rubripes;* Bfl*, Branchiostoma floridae*
